# Cdt1 Is Differentially Targeted for Degradation by Anticancer Chemotherapeutic Drugs

**DOI:** 10.1371/journal.pone.0034621

**Published:** 2012-03-30

**Authors:** Athanasia Stathopoulou, Vassilis Roukos, Chariklia Petropoulou, Panagiotis Kotsantis, Nickolas Karantzelis, Hideo Nishitani, Zoi Lygerou, Stavros Taraviras

**Affiliations:** 1 Department of Physiology, Medical School, University of Patras, Patras, Greece; 2 Department of General Biology, Medical School, University of Patras, Patras, Greece; 3 Department of Biological Signaling, Graduate School of Life Science, University of Hyogo, Hyogo, Japan; National Cancer Institute, United States of America

## Abstract

**Background:**

Maintenance of genome integrity is crucial for the propagation of the genetic information. Cdt1 is a major component of the pre-replicative complex, which controls once per cell cycle DNA replication. Upon DNA damage, Cdt1 is rapidly targeted for degradation. This targeting has been suggested to safeguard genomic integrity and prevent re-replication while DNA repair is in progress. Cdt1 is deregulated in tumor specimens, while its aberrant expression is linked with aneuploidy and promotes tumorigenesis in animal models. The induction of lesions in DNA is a common mechanism by which many cytotoxic anticancer agents operate, leading to cell cycle arrest and apoptosis.

**Methodology/Principal Finding:**

In the present study we examine the ability of several anticancer drugs to target Cdt1 for degradation. We show that treatment of HeLa and HepG2 cells with MMS, Cisplatin and Doxorubicin lead to rapid proteolysis of Cdt1, whereas treatment with 5-Fluorouracil and Tamoxifen leave Cdt1 expression unaffected. Etoposide affects Cdt1 stability in HepG2 cells and not in HeLa cells. RNAi experiments suggest that Cdt1 proteolysis in response to MMS depends on the presence of the sliding clamp PCNA.

**Conclusion/Significance:**

Our data suggest that treatment of tumor cells with commonly used chemotherapeutic agents induces differential responses with respect to Cdt1 proteolysis. Information on specific cellular targets in response to distinct anticancer chemotherapeutic drugs in different cancer cell types may contribute to the optimization of the efficacy of chemotherapy.

## Introduction

Cancer is a complex, multifactorial disease with both genetic and environmental factors involved in its etiology. Despite the complexity, cancer cells exhibit prevailing characteristics that distinguish them from normal cells. Genomic instability is a hallmark of cancer cells, believed to lie at the heart of the acquisition of new traits by cancer cells during neoplastic development. Indeed, around 50% of all tumors exhibit gross chromosomal abnormalities, evident as accumulation of additional copies of genes, genomic regions or whole chromosomes as well as chromosomal rearrangements.

Genomic instability could arise due to the loss of control mechanisms which operate during the normal cell cycle. In eukaryotes, DNA replication needs to be tightly regulated in order to ensure the faithful transmission of the genetic material to the daughter cells. To this end, a process called licensing controls the timely initiation of DNA replication, ensuring that only after passage through mitosis the chromatin becomes competent for a new round of replication. Cdt1 regulates replication licensing by controlling the recruitment of Mini-Chromosome Maintenance proteins (MCMs) onto origins of replication [1]–[3]. Cdt1 is specifically expressed during the G1 phase of the cell cycle [4]–[8] and its function is regulated by multiple independent mechanisms; binding to the inhibitory protein Geminin [6], [9], and degradation through Cdk-SCF^Skp2^ [10]–[12] and Cul4A-DDB1^Cdt2^ pathway [13]–[17]. Overexpression of Cdt1 causes aberrant DNA replication in different experimental systems [18]–[21] and human cells [22], leading to DNA damage and activation of checkpoint pathways [22], [23], while it has been shown that it can also lead to DNA damage without rereplication in non-transformed and quiescent cells [24]. Moreover, Cdt1 is overexpressed in different cancers while recent findings suggest that its expression may participate in the development of the malignant phenotype [23], [25]. Cdt1 is targeted for degradation in response to different types of DNA lesions, and this evolutionarily conserved response has been postulated to constitute an important step in regulating genomic stability and allowing DNA repair [26], [27], [28]. Cdt1 proteolysis requires ubiquitination by the Cul4A-DDB1 ubiquitin ligase and takes place independently of the classic DDR pathway mediated by ATM/ATR and CHK1/CHK2 kinases [15], [16], [26], [27]. Cdt1 ubiquitination has been shown to require interaction with PCNA [14], [15], [16], [29], [30], [31] and the DCAF protein (DDB1- and CUL4-associated factor) Cdt2 [14], [17], [28], [32], [33]. Whereas Cdt1 targeting for degradation in response to UV and γ-irradiation is relatively well understood, little is known about Cdt1 proteolytic degradation in cells treated with commonly used chemotherapeutic anticancer agents, which target DNA. These drugs are among the most effective in clinical practice and have produced significant increases in the survival of patients with cancer when used in combination with drugs that have different mechanisms of actions. However, they show significant limitations, since many patients with cancer either do not respond to the treatment, or develop resistance. In addition, some DNA-damaging agents are toxic and have only a limited therapeutic window. The identification of new cellular targets will help understand the requirements for efficient responses by different types of cancer cells and will provide information for a better understanding of the chemotherapeutic drug's cellular mechanisms of action.

Here we analyze the effect of anticancer agents of the four main classes of DNA targeting chemotherapeutic drugs [34], the alkylating agent methyl methane sulphonate (MMS), cisplatin that forms various DNA adducts, the anti-metabolite 5-FU, the topoisomerase inhibitors etoposide and doxorubicin on targeting the replication factor Cdt1 in different human cancerous cell lines.

## Results

### UV irradiation and alkylating agents target Cdt1 for degradation

**Figure 1:**
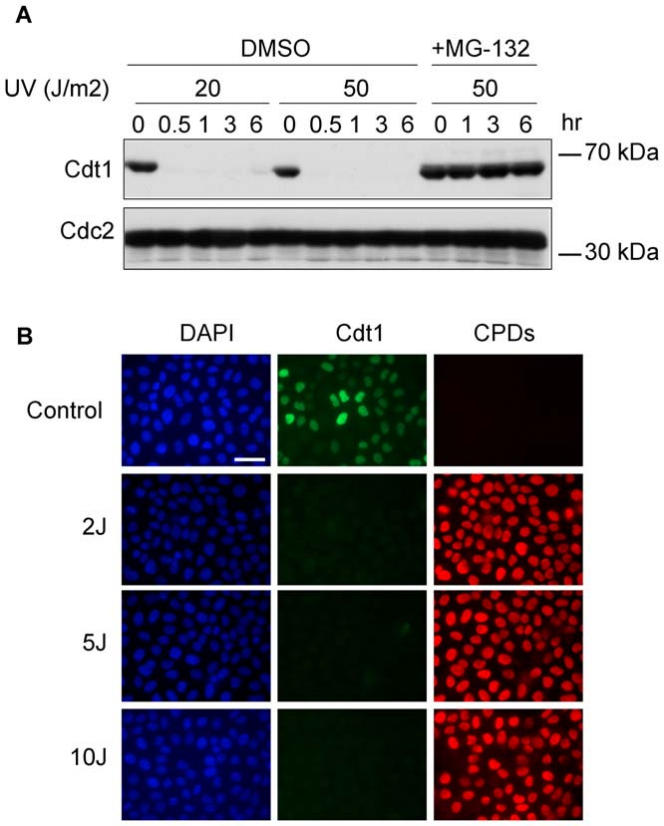
UV irradiation of HeLa cells promotes rapid Cdt1 degradation. (A) HeLa cells were irradiated with 20 and 50 J/m^2^ UV and cells were analyzed after 0.5, 1, 3 and 6 hours. In addition, cells were cultured in the presence of the proteasome inhibitor MG-132 for 2 hr and then irradiated with 50 J/m^2^ UV. Total protein extracts were prepared and subjected to western blot analysis using antibodies against Cdt1. Cdc2 was used as a loading control. (B) HeLa cells were irradiated with 2, 5 and 10 J/m^2^ UV and incubated for 1 hour. Cells were fixed and stained with anti-Cdt1 (green) and anti-CPDs (red) antibodies. DNA was counterstained with DAPI (blue). Scale bars: B, 50 µm.

Cdt1 was previously shown to be targeted for proteolysis following UV treatment of HeLa cells [15], [26], [27], [37]. In accordance with these studies we show that UV irradiation in HeLa cells promotes a rapid Cdt1 degradation within 30 minutes after the induction of the damage which persists up to 6 hours (Figure 1A). Cdt1 degradation was triggered even at low doses of UV irradiation (2 J/m^2^) as depicted by immunofluorescence (Figure 1B) and was reversed in the presence of the proteasome inhibitor MG-132 suggesting that UV-induced Cdt1 targeting for degradation depends on proteasome activity (Figure 1A).

**Figure 2:**
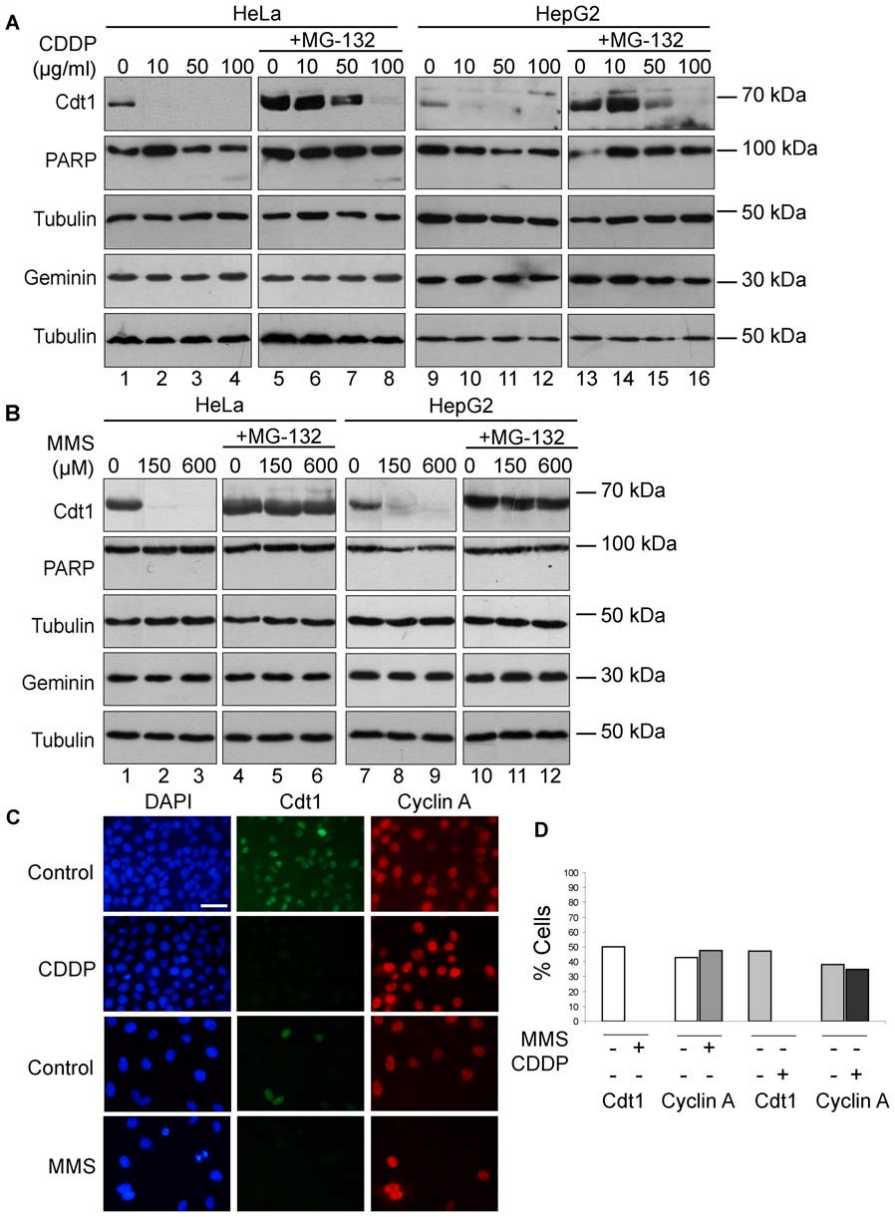
Cdt1 is targeted for proteolysis in response to DNA damage caused by Cisplatin and MMS. HeLa and HepG2 cells were cultured in the presence of Cisplatin (10, 50 and 100 µg/ml) for 6 h (lanes 1–4 and 9–12) or (B) MMS (150 and 600 µM) for 3 h (lanes 1–3 and 7–9) and in the presence of MG-132 (20 µM) (+MG-132) (lanes 5–8 and 13–16 (A) and lanes 4–7 and 10–12 (B)). Cellular protein extracts were prepared and western blot analysis was performed using antibodies against Cdt1, PARP, Geminin and Tubulin as a loading control. (C) HeLa cells cultured in absence or in presence of Cisplatin (50 µg/ml) or MMS (150 µM) were subjected to immunofluorescence analysis using antibodies against Cdt1 and Cyclin A, whereas DNA was stained with DAPI. (D) Percentage of HeLa cells expressing Cdt1 or CyclinA after Cisplatin or MMS treatment. Scale bars: C, 50 µm.

In order to investigate whether routinely used anticancer chemotherapeutic agents activate the Cdt1 proteolysis similar to UV, anticancer agents with distinct mechanisms of action were screened for their ability to target the licensing factor Cdt1 in different human cancerous cell lines. We first examined whether Cdt1 targeting occurs in response to cisplatin known to introduce DNA adducts that mainly result in intrastrand cross-links. HeLa cells were incubated with increasing concentrations of cisplatin and 6 hours upon treatment Cdt1 protein levels were assessed by western blotting (Figure 2A). Cisplatin treatment induces a pronounced reduction of Cdt1 protein levels (Figure 2A, lanes 2–4, left panel), while Geminin protein expression remains unaltered (Figure 2A, left panel). In addition, treatment of HeLa cells with 10, 50 and 100 µg/ml of cisplatin did not result in activation of the apoptotic pathway, as indicated by the intact PARP protein, while PARP cleavage became detectable only in the high concentrations (100 µg/ml) (Figure 2A, left panel). HeLa cells treated in addition to cisplatin with the proteasome inhibitor MG-132 show stabilization of the Cdt1 protein expression (Figure 2A, left panel, lanes 5–8). Similar results were observed when the human hepatocellular liver carcinoma cell line HepG2, was treated with cisplatin, suggesting that cisplatin targets Cdt1 for proteolysis in both cell lines (Figure 2A, right panel).

We then examined whether treatment of HeLa cells with the alkylating agent MMS leads to Cdt1 protein degradation similarly to cisplatin. HeLa cells were treated with increasing concentrations of the specific agent for 3 hours (Figure 2B, left panel) and its protein levels were assessed by western blot. As shown in Figure 2B, Cdt1 is targeted for degradation in response to MMS treatment (lanes 1–3). Similar to what was observed upon UV-irradiation and cisplatin treatment, Cdt1 targeting was proteolysis-dependent, as indicated by the stabilization of Cdt1 protein levels in cells co-treated with the proteasome inhibitor MG-132 (lanes 4–6). A similar effect of MMS treatment on Cdt1 targeting for degradation was observed in HepG2 cells incubated with the same concentrations of MMS, suggesting common ways of regulation in both cell types (Figure 2B, right panel).

In order to assess whether Cdt1 downregulation in response to DNA-damage takes place in cells in the G1 phase of the cell cycle, we employed double immunofluorescence analysis in an asynchronous population of HeLa cells using the expression profile of cyclin A as a specific marker of cells in S, G2 and early M phase of the cell cycle [38]. As shown in Figure 2C and previously reported [4], [7], [15], Cdt1 is expressed specifically in cells in G1 phase and thus its expression is mutually exclusive with cyclin A. Treatment of the cells with either cisplatin or MMS leads to degradation of Cdt1 and absence of Cdt1-specific fluorescent signal, while the percentage of cell expressing cyclin A remained unaffected (Figure 2C, D). These data suggest that Cdt1 degradation upon cisplatin and MMS treatment takes place in cells in G1 phase and is cyclin A-independent. Similar results were obtained in cisplatin-treated synchronized in G1-phase HeLa cells (data not shown). We conclude that cisplatin and MMS lead to proteolysis of Cdt1 in different cancer cells.

### Differential regulation of Cdt1 in response to different topoisomerase II inhibitors

**Figure 3:**
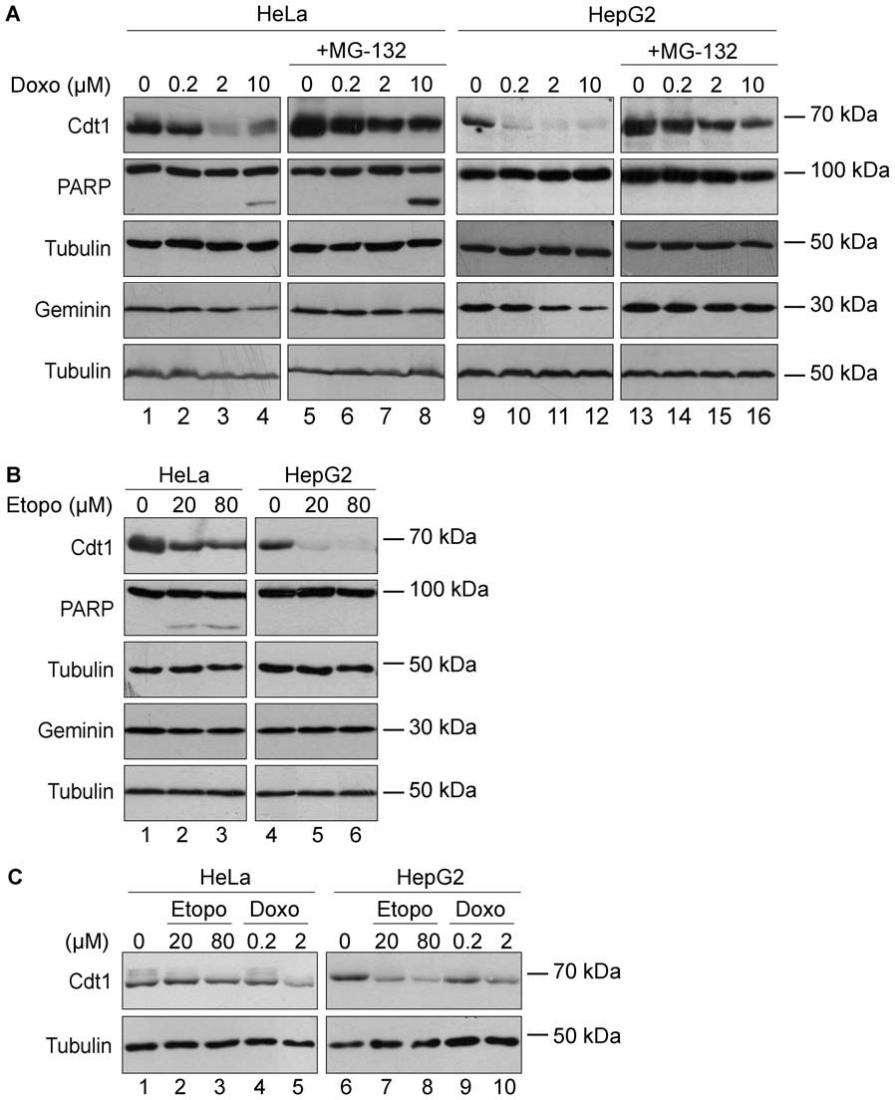
Differential regulation of Cdt1 in response to the topoisomerase inhibitors Doxorubicin and Etoposide. HeLa and HepG2 cells were treated for 6 h with (A) Doxorubicin (0.2, 2 and 10 µM) (Doxo) or (B) Etoposide (20 and 80 µM) (Etopo), in the presence or absence of the proteasome inhibitor MG-132 (+MG-132). Total protein extracts were prepared and subjected to western blot analysis using antibodies against Cdt1, PARP, Geminin and Tubulin. (C) HeLa and HepG2 cells were synchronized in M phase with nocodazole, and subsequently were incubated with Etoposide (20 and 80 µM) (lanes 2–3, 7–8) or Doxorubicin (0.2 and 2 µM) (lanes 4–5, 9–10). Protein extracts were subjected to Western blot analysis using antibodies against Cdt1 and Tubulin.

We next investigated whether Cdt1 targeting for degradation occurs in response to chemotherapeutic agents that promote DNA damage by interfering with the function of topoisomerase II, such as Doxorubicin and etoposide. To this end, HeLa cells were incubated with increasing concentrations of Doxorubicin for 6 hours and western blot analysis was used to assess Cdt1 protein expression levels (Figure 3A). As shown in Figure 3A (left panel), treatment of cells with 0.2, 2 and 10 µM of Doxorubicin resulted in a mild decrease in Cdt1 protein levels while Geminin levels were unaffected (Figure 3A, lanes 2–4). The decrease of Cdt1 protein levels in response to doxorubicin was more profound in doxorubicin-treated HepG2 cells (Figure 3A, lanes 10–12). In both cell lines, co-treatment with the proteasome inhibitor MG-132 resulted in the stabilization of Cdt1 protein levels, implying a proteolysis-dependent Cdt1 targeting. (Figure 3A, lanes 6–8 and 14–16).

Subsequently, HeLa cells were treated with increased amounts of the topoisomerase-II inhibitor etoposide for 6 h and western blot was used to determine Cdt1 protein levels. Cdt1 stability appeared unaffected in HeLa cells treated with etoposide even in high drug concentrations which were able to activate the apoptotic pathway as judged by PARP cleavage (Figure 3B, left panel). However, Cdt1 was profoundly degraded in HepG2 cells treated with etoposide in concentrations that are not efficient to promote apoptosis (Figure 3B, right panel), suggesting a distinct regulation of Cdt1 targeting in response to etoposide treatment between these cell lines (Figure 3B, lanes 5–6).

To investigate in greater detail the observed differential regulation of Cdt1 in response to doxorubicin and etoposide excluding specific cell phase interfering, we assessed the effect of these drugs in Cdt1 stability in cells in the G1 phase of the cell cycle. Since an immunofluorescence-based examination was not possible due to the natural fluorescence of doxorubicin, we synchronized cells in the G1 phase as it is described in materials and methods, while the efficacy of synchronization was tested by immunofluorescence using antibodies against Cdt1 and Cyclin A (data not shown). As shown in Figure 3C, while treatment of synchronized HeLa and HepG2 cells with Doxorubicin resulted in a mild downregulation of Cdt1 at the concentration of 2 µM (Figure 3C, lanes 5 and 10), treatment of HeLa cells with Etoposide does not affect Cdt1 protein levels (Figure 3C, lanes 2, 3). In contrast Cdt1 stability is affected in HepG2 cells in the G1 phase treated with etoposide as shown in Figure 3C (lanes 7, 8).

### 5-Fluouracil and Tamoxifen do not promote Cdt1 degradation

**Figure 4:**
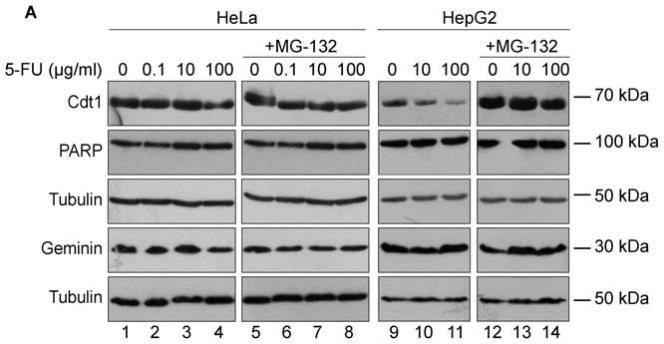
5-Fluoruracil (5-FU) does alter Cdt1 protein expression levels in HepG2 but not in HeLa cells. HeLa and HepG2 cells were incubated for 6 h with 5-FU (0.1, 10 and 100 µg/ml) in the absence (lanes 1–4 and 9–10) or in the presence (lanes 5–8 and 12–14) of MG-132 (20 µM). Protein extracts were analyzed by Western blotting using antibodies against Cdt1, PARP, Geminin and Tubulin.

To address a possible effect of the chemotherapeutic agent 5-FU on Cdt1 targeting upon DNA damage, HeLa cells were treated with the pyrimidine analogue for 6 h and Cdt1 protein levels were asssesed by western blotting. As shown in Figure 4, (lanes 2–4) no alteration of Cdt1 protein levels upon 5-FU treatment was observed. On the contrary, incubation of 5-FU in HepG2 cells resulted in a mild downregulation of Cdt1 expression (Figure 4, lanes 10–11), which was proteolysis-dependent as revealed by stabilization of Cdt1 protein levels in MG-132 treated cells (Figure 4, lanes 13–14). In addition, in accordance with previous results, Geminin protein levels remained unaffected.

**Figure 5:**
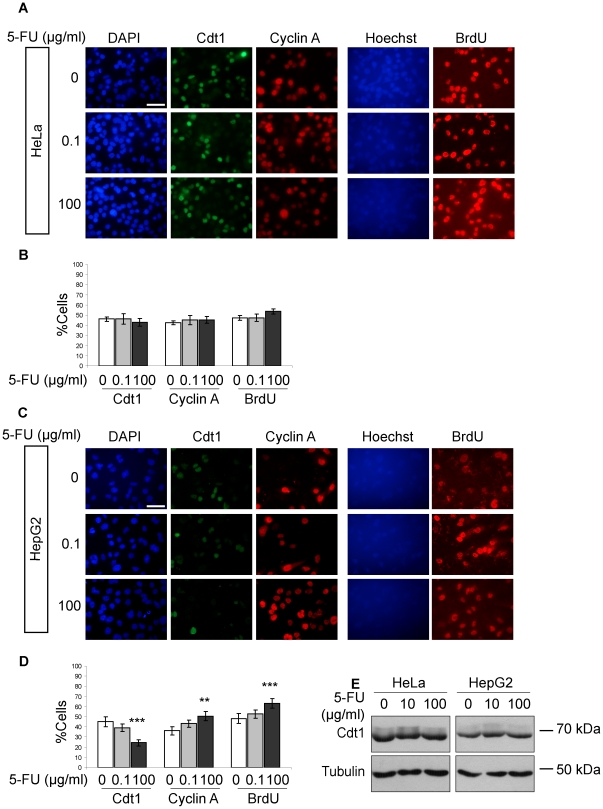
Treatment with 5-Fluoruracil (5-FU) doesn't alter Cdt1 protein expression levels in HeLa or HepG2 cells. Asynchronous HeLa (A) and HepG2 cells (C) were incubated with 5-FU (0.1 and 100 µg/ml) in the presence of BrdU (20 µM, for 1 h). Cells were subjected to immunofluorescence using antibodies against Cdt1, Cyclin A and BrdU. DNA was visualized with DAPI or Hoechst 3258. The percentage of HeLa (B) and HepG2 (D) cells expressing Cdt1, Cyclin A and BrdU in presence of 5-FU, 0.1 µg/ml (grey columns), 100 µg/ml (black columns) and control cells (white columns) is shown; Data are the mean values of the quantifications from at least 3 different experiments from each condition and represent mean ± SD. **p<0.01, ***p<0.001. (E) HeLa and HepG2 cells were synchronized with nocodazole, released to enter G1 phase, and incubated with 5-FU (10 and 100 µg/ml) for 6 hours. Total cell lysates were extracted and subjected to Western blot analysis using antibodies against Cdt1 and Tubulin. Scale bars: A, C, 50 µm.

To further investigate Cdt1 regulation upon 5-FU treatment, the effect of the drug on Cdt1 levels was tested by co-immunolocalisation with cyclin A. An asynchronous population of HeLa cells was treated with 5-FU and double immunofluorescence using antibodies against Cdt1 and Cyclin A was performed (Figure 5A, left panel). In accordance with our previous results, treatment of HeLa cells with 5-FU had no effect on the stability of Cdt1 protein (Figure 5A, left panel and 5B). The percentage of the cells expressing cyclin A was not altered after 5-FU treatment, suggesting that the drug does not arrest cell cycle progression (Figure 5B). In order to mark the percentage of cells undergoing active replication in the presence or absence of 5-FU, HeLa cells were pulsed with the thymidine analogue BrdU which incorporates into DNA during S phase, combined with different concentrations of 5-FU (Figure 5A, right panel). As shown in Figure 5B, the percentage of cells undergoing DNA replication was not altered in the presence of 5-FU, indicating that treatment with 5-FU does not affect the cell cycle profile.

In contrast, the percentage of cells expressing Cdt1 was reduced in HepG2 cells treated with 5-FU by 20% (Figure 5C left panel and 5D). Interestingly, the percentage of the cells expressing cyclin A was increased by approximately 15% (Figure 5C and 5D). Moreover, the percentage of cells incorporating BrdU was also augmented by 15% in HepG2 cells treated with 5-FU (Figure 5C, right panel and 5D), indicating that treatment with 5-FU in this cell line leads to an accumulation of cells in S-phase, where Cdt1 is not expressed.

To investigate the 5-FU effect on Cdt1 targeting in HeLa and HepG2 cells in greater detail, we synchronized both cell lines in G1 phase of the cell cycle and assessed Cdt1 protein levels after treatment with 5-FU. As shown in Figure 5E, Cdt1 protein levels were not affected in synchronized in G1 phase HeLa and HepG2 cells treated with 5-FU, indicating that this drug does not interfere with Cdt1 protein stability.

**Figure 6:**
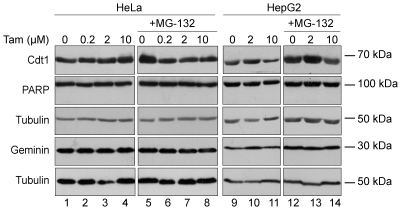
Treatment with Tamoxifen does not affect Cdt1 protein expression levels. HeLa and HepG2 cells were treated with Tamoxifen (0.2, 2 and 10 µM) for 6 h, in absence (lanes 1–4, 9–11) or in presence (lanes 5–8, 12–14) of MG-132. Cells were harvested, protein extracts were prepared and subjected to Western blot analysis using antibodies against Cdt1 and Tubulin as a loading control.

These data suggest that different chemotherapeutic agents that induced DNA damage show differential response on Cdt1 targeting for proteolysis. To explore the effect of a chemotherapeutic drug that does not induce DNA damage on Cdt1 stability, we treated HeLa and HepG2 cells with increased concentrations of the estrogen antagonist Tamoxifen (Tam). As illustrated in Figure 6, Cdt1 protein expression remains unaffected after Tam treatment in both HeLa and HepG2 cells, suggesting that Cdt1 degradation is regulated by chemotherapeutic agents that induce DNA damage only.

### Cdt1 degradation in response to chemotherapeutic agents depends on PCNA

**Figure 7:**
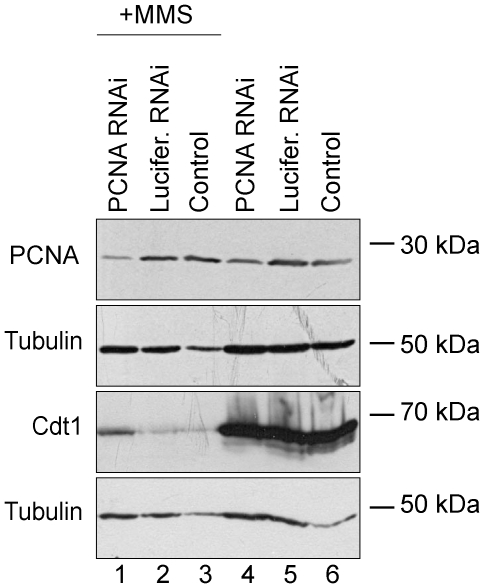
PCNA is involved in the Cdt1 proteolysis pathway. HeLa cells were transfected with 100 nM siRNAs for PCNA (PCNA RNAi) and Luciferase (Lucifer. RNAi) for 72 h. Subsequently, cells were either uncultured or cultured in the presence of MMS (600 µM) (lanes 1–3) for 3 h before cell lysis. Total cell lysates were prepared and analyzed by Western blot using antibodies against PCNA, Cdt1, and Tubulin.

Previous studies revealed that Cdt1 targeting for proteolysis upon DNA damage requires the ubiquitin ligase Cul4A-Ddb1^Cdt2^ and interaction with PCNA [14], [15], [16], [28], [29], [30], [32]. To investigate whether the same pathway targets Cdt1 for degradation in response to DNA damage caused by the drugs used in this study, we silenced PCNA expression using siRNA technology. As shown in Figure 7, knock-down of PCNA expression in HeLa cells treated with MMS leads to a corresponding rescue of Cdt1 degradation compared to siRNA for Luciferase –MMS-treated cells (compare lanes 1 and 2). These results indicate that PCNA is required for Cdt1 degradation upon DNA damage caused by MMS.

## Discussion

One of the current approaches to modern cancer treatment is to identify cancer-specific molecular targets against which drugs can be developed. However, cancer is a highly complex disease, showing genetic variability not only between different cancer types, but also between patients having the same cancer type and even different cells within the same tumour. The diversity of cancer calls for identification of drugs aiming against multiple targets to ensure efficient responses by different types of cancer cells. In addition, discovering new cellular targets of the commonly used chemotherapeutic agents will help understanding their cellular mechanisms of action. Here we explore the effects of anticancer agents with distinct mechanisms of action on the targeting of the replication factor Cdt1 in different human cancerous cell lines, simulating the effect of these drugs in the activation of Cdt1-dependent checkpoint in different cancer types.

Cisplatin is a platinum-based drug that distorts the structure of the DNA duplex, activating the NER (Nucleotide Excision Repair) pathways, the major pathway responsible for the removal of cisplatin–DNA adducts. The treatment with cisplatin activates cell cycle checkpoints through the activation of ATM/ATR and the downstream Chk2 and Chk1 kinases [39] and modulates several signal transduction pathways such as the AKT (v-akt murine thymoma viral oncogene homologue) pathway, c-ABL (v-abl Abelson murine leukaemia viral oncogene homologue 1), p53, MAPK (mitogen-activated protein kinase)/JNK (c-Jun NH2-terminal kinase)/ERK (extracellular signal-regulated kinase), pathways which interfere with cisplatin's cytotoxicity [reviewed in [40]]. Here, we show that Cdt1 is targeted for proteolysis-dependent degradation in response to cisplatin, in both the cervical carcinoma cell line HeLa and the hepatoma cell line HepG2, suggesting that this drug is able to activate the Cdt1-dependent checkpoint in different cancer cells. Interestingly, while cisplatin induces checkpoint activation through the ATM/ATR pathway, Cdt1 degradation in response to DNA damage is ATM/ATR-independent [26].

Topoisomerase II (TOP2) is the target of several important classes of anticancer drugs, including the epipodophyllotoxin etoposide and the anthracycline doxorubicin [41]. As these drugs are highly active anticancer agents in many different clinical settings, we asked whether the replication protein Cdt1 is targeted for degradation upon treatment. Surprisingly, Cdt1 shows differential regulation in response to the different topoisomerase II poisons. The treatment of both HeLa and HepG2 cells with doxorubicin results in the activation of the Cdt1-dependent checkpoint, although this targeting was less pronounced than following cisplatin treatment. Similarly, etoposide treatment results in Cdt1 degradation in HepG2 cells. In contrast, Cdt1 is not targeted in HeLa cells treated with etoposide, suggesting a differential Cdt1 targeting after treatment with different topo2 drugs and between different cell lines. Interestingly, doxorubicin and etoposide belong to different Topoisomerase II poison categories in respect to their ability to intercalate or not to DNA. Doxorubicin is able to intercalate to DNA and notably has a range of effects on cells, in addition to inhibition of TOP2, such as to production of free radicals, membrane damage and induction of protein–DNA crosslinks [41]. In contrast, etoposide belongs to non-intercalating Topo2 poisons believed to induce damage through protein–drug interactions that have key roles in the ability of TOP2 poisons to trap TOP2 covalent complexes [42], [43]. The cell-type specificity following etoposide treatment may be dependent on a cell-type specific ability of the poison to trap TOP2 covalent complexes or may reflect cell type specific differences in the cell cycle machinery and/or the repair pathways. Our data suggest that etoposide and doxorubicin could be used in a combinatorial antitumorigenic therapy in order to effectively target Cdt1 degradation and this chemotherapeutic scheme might target more efficiently cell proliferation of different cell types.

Our results indicate that Cdt1 degradation in response to chemotherapeutic agents takes place in G1 phase of the cell cycle and is cyclinA-independent [15], [26]. We would therefore anticipate that agents that act in different phases of the cell cycle would not affect Cdt1 stability upon genotoxic stress. Indeed, the treatment of cells with the pyrimidine nucleotide analogue 5-Fluoruracil (5-FU), which as an antimetabolite drug directly affects the supply of dNTPs to replicative polymerases and thus acts during S phase of the cell cycle, did not induce Cdt1 degradation in both synchronized in G1 phase HeLa and HepG2 cells. In support of this, Cdt1 was targeted for degradation in response to the alkylating agent MMS and the platinum-based drug cisplatin, which modify the DNA structure and induce DNA damage during all the phases of the cell cycle, including G1.

The estrogen receptor antagonist Tamoxifen, widely used as a chemotherapeutic drug for breast cancer, does not induce DNA damage. As expected, in cells treated with Tamoxifen, Cdt1 was not targeted for degradation, indicating that Cdt1 proteolysis is activated specifically upon DNA damage by chemotherapeutic drugs that act in G1.

Previous studies suggest that the Cdt1 degradation pathway upon DNA damage induced by UV and ionizing radiation requires direct interaction with PCNA and ubiquitination by the Cul4A-Ddb1^Cdt2^ ubiquitin ligase [13], [15], [16], [26], [27], [30]. Whether the same pathway targets Cdt1 in response to chemotherapeutic anticancer agents is not known. Our experiments of knocking down the expression of PCNA using siRNA suggest that PCNA is required for the degradation of Cdt1 in response to MMS, indicating that similar mechanisms to preserve genomic integrity in response to different insults.

Cdt1 expression is increased in colon and non-small-cell lung carcinomas [25], [44], [45]. Moreover, Cdt1 overexpression has been linked with increased tumor growth values, aneuploidy and worst prognosis of non-small-cell lung carcinomas patients when combined with mutations in p53 [25], [45]. This is in accordance with experiments that show that Cdt1 expressing cells formed tumors in nude mice and furthermore transgenic mice that overexpress Cdt1 specifically in T cells developed lymphoblastic lymphomas when crossed with p53 null mice [46], [47]. Moreover Liontos et al., have suggested that Cdt1 overexpression could play a role in cancer development as its overexpression can occur early in premalignant states and participate in tumor development [23]. Recent studies in cancer biology have revealed a rare population of cells that can be found in tumors, have stem cell-like properties, survive after drug treatment or surgical removal of the tumor, and can reinitiate the tumor [48], [49]. Several studies have tried to shed light on the biology of these cancer stem cells [50], [51], but still they are only poorly understood. Since these cells are actively replicating cells, anticancer agents that induce the degradation of the licensing factor Cdt1 could be used in order to specifically eliminate this cell population.

In conclusion, our study suggests that genotoxic therapies used routinely against cancer differentially affect Cdt1-dependent degradation and consequently licensing regulation. Information about the specific cellular targets in response to distinct anticancer chemotherapeutic drugs in different cancer cell types will contribute to the optimization of the efficacy of chemotherapy through a more accurate classification and a better understanding of their mechanism of action. Combining the best chemotherapeutic action with specific targets in cellular pathways offers a powerful new approach to cancer treatment that might counteract the many ways that human cells can become drug-resistant, while Cdt1 targeting might be *per se* essential as a factor promoting tumor development.

## Materials and Methods

### Cell culture and DNA damage induction

HeLa and HepG2 cells (ATCC) were cultured in Dulbecco's Modified Eagle's (DMEM) medium (GIBCO) with 10% (v/v) Fetal Bovine Serum (GIBCO), 1% (v/v) penicillin/streptomycin (GIBCO) at 37°C and 5% CO_2_ atmosphere. Subconfluent HeLa and HepG2 cell cultures were incubated with Methyl Methane Sulfonate (MMS, Aldrich Chem. Co.) for 3 h, and the chemotherapeutic agents Cisplatin [cis-diamminedichloroplatinum(II), CDDP], Doxorubicin Hydrochloride, 5-Fluorouracil, Etoposide and Tamoxifen at the indicated concentrations for 3 h. The chemotherapeutic drugs were purchased from Sigma and prepared from stock solutions in dimethyl sulfoxide (DMSO), except MMS which was prepared from stock solution in sterile H_2_O. Where indicated, cells were incubated with 20 µM MG-132 (Calbiochem) for 3 h before the end of drug treatment. UV-irradiation was carried out at 2–50 J/m^2^ using a Stratalinker.

### Cell synchronization

To synchronize HeLa cells in mitosis, exponentially growing cells were treated with 5 ng/ml Nocodazole (Sigma) for 16 h [35]. Mitotic cells were collected by shake-off in cold PBS, washed twice in PBS, and further cultured for 2–3 hours in complete DMEM without nocodazole, so as to proceed in G1 phase before drug treatment. The same protocol was followed for synchronizing HepG2 cells.

### Western blotting

Total cell lysates, prepared by lysing cell pellets directly in SDS-page loading buffer, were subjected to electrophoresis in 6% acrylamide gels for Cdt1 and PARP, 10% for PCNA and 15% for Geminin and transferred to PVDF membranes (Millipore). Immunodetection was performed using affinity purified polyclonal antibodies against Cdt1 (1∶2.000) [4] and Geminin (1∶2.000) [7], PARP (BD Pharmigen, 1∶2.000), and monoclonal anti-tubulin (Sigma, 1∶20.000), anti-PCNA (Santa Cruz Biotechnology, 1∶500)and anti-Cdc2 (1∶5.000) [15].

### Immunofluorescence

For immunofluorescence, cells were grown on glass slides and after drug treatment or UV irradiation they were fixed in 4% formaldehyde for 10 min, washed twice with PBS, permeabilized with 0.3% TritonX-100 in PBS and then washed three times with PBS. Cells were treated with blocking buffer (3% BSA, 10% fetal bovine serum in PBS) for 1 hour and incubated with primary antibodies overnight in a wet chamber. Cells were washed in PBS containing 0.1% Tween three times and incubated for 1 h with fluorescently labeled secondary antibodies, Alexa-Fluor-568 goat anti-mouse IgG and Alexa-Fluor-488 goat anti-mouse IgG. After washing, DNA was stained with DAPI (Vector Laboratories). Polyclonal antibodies used against Cdt1 (1∶600) and Geminin (1∶1500) were previously described [4], [7], [36], and monoclonal anti-Cyclin A (Neomarkers, 1∶40). Thymidine dimmers were visualized using an antibody directed against CPDs (Kamiya Biomedical Company, 1∶500).

### BrdU staining

Asynchronous growing cells were pulsed with 20 µM BrdU (5-bromo-2-deoxyuridine) (Sigma) for 1 h followed by fixation in 4% PFA for 10 min. Then cells were washed twice with PBS, permeabilized with 0.3% TritonX-100 in PBS and then washed three times with PBS. DNA was denatured for 1 h with 2 N HCl, and then cells were washed with 0,1 M Tris-HCl pH 8,8 and three times with PBS. Cells were treated with blocking buffer (3% BSA, 10% fetal bovine serum in PBS) for 1 h and incubated with primary rat anti-BrdU (Oxford Biotechnology, 1∶80) overnight in a wet chamber. Cells were washed in PBS containing 0.1% Tween three times and incubated with fluorescently labeled secondary antibody, Alexa-Fluor-568 goat anti-rat IgG. After washing, DNA was visualized with Hoechst.

### RNAi experiments

For RNAi experiments, we used human PCNA *siGENOME SMART* pool which was synthesized and obtained from Dharmacon, Inc., Lafyette. CO. As a control, siRNA for Luciferase (MWG, Biotech) was used. HeLa cells were plated in 30 mm dish and transfected with 100 nM siRNA on the following day (when cells were 30% confluent) using DharmaFECT, following DharmaFECT siRNA transfection protocol (Dharmacon, Inc., Lafyette. CO). At 72 h post transfection, cells were collected and protein extracts were analyzed by WB. When indicated, cells were treated with 600 µM MMS for 3 h and before collecting cells.

### Image acquisition and data analysis

Images were acquired with a Nikon Eclipse TE2000-U microscope and collected with a Nikon Digital Sight DS-L1 camera. All the data presented here are obtained from at least three different experiments. The results are given as mean ± SD and the statistical significance was based on the Student's t-test, with *P<0.05, **P<0.01 and ***P<0.001.

## References

[1] Maiorano D, Moreau J, Mechali M (2000). XCDT1 is required for the assembly of pre-replicative complexes in Xenopus laevis.. Nature.

[2] Nishitani H, Lygerou Z, Nishimoto T, Nurse P (2000). The Cdt1 protein is required to license DNA for replication in fission yeast.. Nature.

[3] Wong PG, Glozak MA, Cao TV, Vaziri C, Seto E (2010). Chromatin unfolding by Cdt1 regulates MCM loading via opposing functions of HBO1 and HDAC11-geminin.. Cell Cycle.

[4] Nishitani H, Taraviras S, Lygerou Z, Nishimoto T (2001). The human licensing factor for DNA replication Cdt1 accumulates in G1 and is destabilized after initiation of S-phase.. J Biol Chem.

[5] Sakaue-Sawano A, Kurokawa H, Morimura T, Hanyu A, Hama H (2008). Visualizing spatiotemporal dynamics of multicellular cell-cycle progression.. Cell.

[6] Wohlschlegel JA, Dwyer BT, Dhar SK, Cvetic C, Walter JC (2000). Inhibition of eukaryotic DNA replication by geminin binding to Cdt1.. Science.

[7] Xouri G, Lygerou Z, Nishitani H, Pachnis V, Nurse P (2004). Cdt1 and geminin are down-regulated upon cell cycle exit and are over-expressed in cancer-derived cell lines.. Eur J Biochem.

[8] Xouri G, Squire A, Dimaki M, Geverts B, Verveer PJ (2007). Cdt1 associates dynamically with chromatin throughout G1 and recruits Geminin onto chromatin.. Embo J.

[9] Tada S, Li A, Maiorano D, Mechali M, Blow JJ (2001). Repression of origin assembly in metaphase depends on inhibition of RLF-B/Cdt1 by geminin.. Nat Cell Biol.

[10] Li X, Zhao Q, Liao R, Sun P, Wu X (2003). The SCF(Skp2) ubiquitin ligase complex interacts with the human replication licensing factor Cdt1 and regulates Cdt1 degradation.. J Biol Chem.

[11] Liu E, Li X, Yan F, Zhao Q, Wu X (2004). Cyclin-dependent kinases phosphorylate human Cdt1 and induce its degradation.. J Biol Chem.

[12] Sugimoto N, Tatsumi Y, Tsurumi T, Matsukage A, Kiyono T (2004). Cdt1 phosphorylation by cyclin A-dependent kinases negatively regulates its function without affecting geminin binding.. J Biol Chem.

[13] Arias EE, Walter JC (2006). PCNA functions as a molecular platform to trigger Cdt1 destruction and prevent re-replication.. Nat Cell Biol.

[14] Higa LA, Banks D, Wu M, Kobayashi R, Sun H (2006). L2DTL/CDT2 interacts with the CUL4/DDB1 complex and PCNA and regulates CDT1 proteolysis in response to DNA damage.. Cell Cycle.

[15] Nishitani H, Sugimoto N, Roukos V, Nakanishi Y, Saijo M (2006). Two E3 ubiquitin ligases, SCF-Skp2 and DDB1-Cul4, target human Cdt1 for proteolysis.. Embo J.

[16] Senga T, Sivaprasad U, Zhu W, Park JH, Arias EE (2006). PCNA is a cofactor for Cdt1 degradation by CUL4/DDB1-mediated N-terminal ubiquitination.. J Biol Chem.

[17] Sansam CL, Shepard JL, Lai K, Ianari A, Danielian PS (2006). DTL/CDT2 is essential for both CDT1 regulation and the early G2/M checkpoint.. Genes Dev.

[18] Arias EE, Walter JC (2005). Replication-dependent destruction of Cdt1 limits DNA replication to a single round per cell cycle in Xenopus egg extracts.. Genes Dev.

[19] Davidson IF, Li A, Blow JJ (2006). Deregulated replication licensing causes DNA fragmentation consistent with head-to-tail fork collision.. Mol Cell.

[20] Thomer M, May NR, Aggarwal BD, Kwok G, Calvi BR (2004). Drosophila double-parked is sufficient to induce re-replication during development and is regulated by cyclin E/CDK2.. Development.

[21] Zhong W, Feng H, Santiago FE, Kipreos ET (2003). CUL-4 ubiquitin ligase maintains genome stability by restraining DNA-replication licensing.. Nature.

[22] Vaziri C, Saxena S, Jeon Y, Lee C, Murata K (2003). A p53-dependent checkpoint pathway prevents rereplication.. Mol Cell.

[23] Liontos M, Koutsami M, Sideridou M, Evangelou K, Kletsas D (2007). Deregulated overexpression of hCdt1 and hCdc6 promotes malignant behavior.. Cancer Res.

[24] Tatsumi Y, Sugimoto N, Yugawa T, Narisawa-Saito M, Kiyono T (2006). Deregulation of Cdt1 induces chromosomal damage without rereplication and leads to chromosomal instability.. J Cell Sci.

[25] Petropoulou C, Kotantaki P, Karamitros D, Taraviras S (2008). Cdt1 and Geminin in cancer: markers or triggers of malignant transformation?. Front Biosci.

[26] Higa LA, Mihaylov IS, Banks DP, Zheng J, Zhang H (2003). Radiation-mediated proteolysis of CDT1 by CUL4-ROC1 and CSN complexes constitutes a new checkpoint.. Nat Cell Biol.

[27] Hu J, McCall CM, Ohta T, Xiong Y (2004). Targeted ubiquitination of CDT1 by the DDB1-CUL4A-ROC1 ligase in response to DNA damage.. Nat Cell Biol.

[28] Ralph E, Boye E, Kearsey SE (2006). DNA damage induces Cdt1 proteolysis in fission yeast through a pathway dependent on Cdt2 and Ddb1.. EMBO Rep.

[29] Havens CG, Walter JC (2009). Docking of a specialized PIP Box onto chromatin-bound PCNA creates a degron for the ubiquitin ligase CRL4Cdt2.. Mol Cell.

[30] Hu J, Xiong Y (2006). An evolutionarily conserved function of proliferating cell nuclear antigen for Cdt1 degradation by the Cul4-Ddb1 ubiquitin ligase in response to DNA damage.. J Biol Chem.

[31] Guarino E, Shepherd ME, Salguero I, Hua H, Deegan RS (2011). Cdt1 proteolysis is promoted by dual PIP degrons and is modulated by PCNA ubiquitylation.. Nucleic Acids Res.

[32] Jin J, Arias EE, Chen J, Harper JW, Walter JC (2006). A family of diverse Cul4-Ddb1-interacting proteins includes Cdt2, which is required for S phase destruction of the replication factor Cdt1.. Mol Cell.

[33] Shibata E, Abbas T, Huang X, Wohlschlegel JA, Dutta A (2011). Selective ubiquitylation of p21 and Cdt1 by UBCH8 and UBE2G ubiquitin-conjugating enzymes via the CRL4Cdt2 ubiquitin ligase complex.. Mol Cell Biol.

[34] Zhou BB, Bartek J (2004). Targeting the checkpoint kinases: chemosensitization versus chemoprotection.. Nat Rev Cancer.

[35] Gasnereau I, Ganier O, Bourgain F, de Gramont A, Gendron MC (2007). Flow cytometry to sort mammalian cells in cytokinesis.. Cytometry A.

[36] Spella M, Britz O, Kotantaki P, Lygerou Z, Nishitani H (2007). Licensing regulators Geminin and Cdt1 identify progenitor cells of the mouse CNS in a specific phase of the cell cycle.. Neuroscience.

[37] Kondo T, Kobayashi M, Tanaka J, Yokoyama A, Suzuki S (2004). Rapid degradation of Cdt1 upon UV-induced DNA damage is mediated by SCFSkp2 complex.. J Biol Chem.

[38] Pines J, Hunter T (1990). Human cyclin A is adenovirus E1A-associated protein p60 and behaves differently from cyclin B.. Nature.

[39] Wang J, Pabla N, Wang CY, Wang W, Schoenlein PV (2006). Caspase-mediated cleavage of ATM during cisplatin-induced tubular cell apoptosis: inactivation of its kinase activity toward p53.. Am J Physiol Renal Physiol.

[40] Wang D, Lippard SJ (2005). Cellular processing of platinum anticancer drugs.. Nat Rev Drug Discov.

[41] Nitiss JL (2009). Targeting DNA topoisomerase II in cancer chemotherapy.. Nat Rev Cancer.

[42] Bender RP, Jablonksy MJ, Shadid M, Romaine I, Dunlap N (2008). Substituents on etoposide that interact with human topoisomerase IIalpha in the binary enzyme-drug complex: contributions to etoposide binding and activity.. Biochemistry.

[43] Wilstermann AM, Bender RP, Godfrey M, Choi S, Anklin C (2007). Topoisomerase II - drug interaction domains: identification of substituents on etoposide that interact with the enzyme.. Biochemistry.

[44] Bravou V, Nishitani H, Song SY, Taraviras S, Varakis J (2005). Expression of the licensing factors, Cdt1 and Geminin, in human colon cancer.. Int J Oncol.

[45] Karakaidos P, Taraviras S, Vassiliou LV, Zacharatos P, Kastrinakis NG (2004). Overexpression of the replication licensing regulators hCdt1 and hCdc6 characterizes a subset of non-small-cell lung carcinomas: synergistic effect with mutant p53 on tumor growth and chromosomal instability–evidence of E2F-1 transcriptional control over hCdt1.. Am J Pathol.

[46] Arentson E, Faloon P, Seo J, Moon E, Studts JM (2002). Oncogenic potential of the DNA replication licensing protein CDT1.. Oncogene.

[47] Seo J, Chung YS, Sharma GG, Moon E, Burack WR (2005). Cdt1 transgenic mice develop lymphoblastic lymphoma in the absence of p53.. Oncogene.

[48] Ponti D, Costa A, Zaffaroni N, Pratesi G, Petrangolini G (2005). Isolation and in vitro propagation of tumorigenic breast cancer cells with stem/progenitor cell properties.. Cancer Res.

[49] Sharma SV, Lee DY, Li B, Quinlan MP, Takahashi F (2010). A chromatin-mediated reversible drug-tolerant state in cancer cell subpopulations.. Cell.

[50] Achuthan S, Santhoshkumar TR, Prabhakar J, Nair SA, Pillai MR (2011). Drug-induced senescence generates chemoresistant stemlike cells with low reactive oxygen species.. J Biol Chem.

[51] Musch T, Oz Y, Lyko F, Breiling A (2010). Nucleoside drugs induce cellular differentiation by caspase-dependent degradation of stem cell factors.. PLoS One.

